# Effects of energetic restriction diet on butyrylcholinesterase in obese women from southern Brazil – A longitudinal study

**DOI:** 10.1590/2359-3997000000268

**Published:** 2017-06-14

**Authors:** Willian dos Santos, Luciane Viater Tureck, Louise Farah Saliba, Caroline Schovanz Schenknecht, Débora Scaraboto, Ricardo Lehtonen R. Souza, Lupe Furtado-Alle

**Affiliations:** 1 Laboratório de Polimorfismos e Ligação Departamento de Genética Universidade Federal do Paraná Curitiba PR Brasil Laboratório de Polimorfismos e Ligação, Departamento de Genética, Universidade Federal do Paraná (UFPR), Curitiba, PR, Brasil; 2 Departamento de Educação Universidade Tecnológica Federal do Paraná Curitiba PR Brasil Departamento de Educação, Universidade Tecnológica Federal do Paraná (UTFPR), Curitiba, PR, Brasil

**Keywords:** Obesity, dietetic intervention, butyrylcholinesterase activity, BChE molecular forms, lipid metabolism

## Abstract

**Objective:**

Butyrylcholinesterase (BChE) activity has been associated with obesity, lipid concentrations, and CHE2 locus phenotypes. This, the aim of this study was to evaluate the effects of an energetic restriction diet intervention on anthropometrical and biochemical variables and on absolute and relative BChE activity in CHE2 C5+ and CHE2 C5- individuals.

**Subjects and methods:**

One hundred eleven premenopausal obese women from Southern Brazil participated in an energetic restriction diet intervention (deficit of 2500 kJ/day) for 8 weeks. Their anthropometric and biochemical parameters were evaluated before and after the intervention. Plasma BChE activity was measured, and BChE bands in plasma and CHE2 locus phenotypes were detected by electrophoresis.

**Results:**

The dietetic intervention decreased anthropometric and biochemical parameters as well as absolute BChE activity and relative activity of the G4 band. The CHE2 C5+ phenotype presented a different effect when compared with the CHE2 C5- phenotype. The CHE2 C5+ phenotype showed an effect in absolute BChE activity and in the relative activity of the G4 form, maintaining higher BChE activity regardless of the metabolic changes.

**Conclusion:**

In our study, 8 weeks was not sufficient time to lower the body mass index to normal, but it was enough to significantly reduce the absolute BChE activity, which became similar to the levels in nonobese individuals. CHE2 C5+ individuals were resistant to the decrease in BChE activity compared to CHE2 C5- individuals. This shows that the diet did not affect the CHE2 and G4 fraction complex and that the products of the CHE2 locus in association with BChE have a role in energy metabolism, maintaining high levels of enzymatic activity even after dietary intervention.

## INTRODUCTION

Human butyrylcholinesterase (BChE; EC 3.1.1.8) is an esterase encoded by the *BCHE* gene (3q26.1-q26.2) (1), synthesized in the liver, and distributed to several parts of the organism (2). Despite the fact that BChE’s main function and its natural substratum remain unknown, it has been related to lipid metabolism and has been associated with body mass index (BMI), waist-hip ratio, waist circumference, weight, and cholesterol and triglyceride levels (3-8). Plasmatic BChE is found in 5 different molecular structures: G1 (monomers), G1-A (monomers linked to albumin), G2 (dimers), G3 (trimers), and G4 (tetramers) (9).

Chemically, G2 is formed by a disulfide bond between 2 G1 monomers (10), and G4 is formed by 2 G2 dimers bonded by noncovalent interactions (11). The organization and stabilization of G4 is mediated by lamellipodin proline rich peptides (12-14).

In addition to these 4 basic BChE molecular forms, a protein can bind to the G4 tetramers and originate the C5 band, determining 2 phenotypes: CHE2 C5+ and CHE2 C5-, characterized by the presence and the absence of the extra C5 band, respectively (15). The frequency of the C5 band in a population sample of Southern Brazil was 10.3% (16). Although the protein associated with G4 to form the C5 band is not known yet, its coding gene has been mapped to chromosome 2q33-35 (*CHE2* locus). CHE2 C5+ individuals show mean BChE activity approximately 30% higher than CHE2 C5- individuals (15,17). Despite the fact that the higher enzyme activity is related to weight, obesity, and BMI, CHE2 C5+ individuals presented a lower BMI and weight when compared with CHE2 C5- individuals, suggesting that the presence of the C5 complex has a protective effect against obesity (15,18).

Considering the evidence connecting BChE with obesity, the aim of this work is to investigate the effects of an energetic restriction diet on total and relative BChE activity in relation to the CHE2 C5 phenotype in obese women from Southern Brazil.

## SUBJECTS AND METHODS

### Subjects

This study was previously approved by the ethical committee of the Pontifical Catholic University of Paraná (PUC-PR), under registration 0005306/11.

Women living in Curitiba and neighboring cities were invited to participate in this study via local radio and television ads, with the aim to reduce weight. Initially, we had a group of 199 obese women; at the end of the study, 111 completed the dietetic intervention. Only this group was statistically analyzed.

All the women in the study had a BMI ≥ 30, being an inclusion criterion, along with being 20 years old or older, being in their reproductive period (not in menopause), not being pregnant, and not lactating. The women were excluded when they were already participating in a diet; using weight control medicaments; had a confirmed diagnosis of diabetes I, noncontrolled hypertension, hypothyroidism, or renal chronic disease; had undergone stomach reduction surgery; had been a vegetarian; and did not have the availability to attend the meetings at PUC-PR on Saturdays. The women who agreed to participate and who were in agreement with the study criteria read, accepted, and signed the terms of informed consent (IC).

The 111 women who started and concluded the study had predominantly Euro-Brazilian ancestry (self-declared). Their ages ranged from 20 to 50 years (all in the premenopausal stage), and the predominant age range was 30-39 years (45% of participants). During the study, 70.4% of the women were employed (19).

In the preintervention phase, 56% of the participants were class I obese (30-34.9 kg/m^2^), 24.8% were class II obese (35-39.9 kg/m^2^), and 19.2% were class III obese (≥ 40 kg/m^2^) (19).

### Study design and energetic restriction diet

The study design was longitudinal, or a quasi-experimental intervention, measured using an analysis of variables before and after the dietetic intervention. The diet comprised 9 weeks of energetic restriction, and the eating habits before the intervention were documented in an interview, during which participants were instructed to follow the diet. It was made an adequacy of the intake of macronutrients before the intervention to the current recommendations (20). The nutritional orientation was set up to provide a deficit of 2500 kJ/day, and the diet models were developed from the adapted protocol of diet intervention of Nugenob (21) and with the recommendations of the American Dietetic Association (22). The nutritional percentage of each macronutrient was recommended as follows: the energy from fat was in the range of 20% to 35%, from carbohydrates in the range of 45% to 65%, and from proteins in the range of 10% to 35% (23).

### Biochemical and anthropometric parameters

Blood samples were collected after the agreement of participation and instruction of 8 to 12 hours of fasting at 2 instances: before and after the dietetic intervention. Biochemical parameters, total cholesterol (TC), high density lipoprotein cholesterol (HDL-C), low density lipoprotein cholesterol (LDL-C), and triglycerides (TG) were obtained by standard automated methods.

Weight and height were measured with an accuracy of 0.1 kg and 0.1 cm, respectively. BMI, waist circumference (WC), and abdominal circumference (AC) were also measured. The abdominal-stature ratio (ASR) and waist-stature ratio (WSR) were obtained from these measurements.

### BChE activity analysis and detection of BChE bands

Plasma BChE activity was measured using the protocol of Dietz and cols. (24) modified by Evans and Wroe (25) *CHE2* locus phenotypes were identified by acid agar gel electrophoresis (pH: 6.40) (26).

The detection of BChE bands in plasma was made according to Boberg and cols. (27). The relative intensity (RI) of each band was measured by KODAK 1D Image Analysis Software, and the relative activity (RA) of each band was the result of multiplication of the total BChE plasma activity by the RI of each band. Samples without any detectable BChE band were excluded from the analysis.

### Statistics

The phenotype frequencies of CHE2 C5- and CHE2 C5+ were obtained by direct counting. The comparisons between means (biochemical and anthropometric parameters and BChE activity) were performed by *t* test (parametric variables) or by Mann-Whitney test (nonparametric and independent variables). Multiple regression analysis was performed to verify the interaction between variables. The probability value for the comparative tests was considered significant at *p* < 0.05 (5%).

## RESULTS

The anthropometric variables WC, AC, and BMI suffered a significant decrease after the intervention, as did the mean plasma absolute BChE activity (Table 1).

**Table 1:** Means (± SE) of the parameters before and after the dietetic intervention and their comparisons (P)

Parameters	Before	After	P
Weight (kg)	90.76 ± 14.62	88.93 ± 15.19	0.29
Waist circumference (cm)	95.70 ± 9.88	91.36 ± 13.24	0.0008^a^
Abdominal circumference (cm)	109.44 ± 11.55	102.05 ± 10.84	< 0.0001^a^
BMI (kg/m^2^)	35.14 ± 5.32	33.81 ± 6.04	< 0.0001^a^
TC (mg/dL)	192.94 ± 37.98	192.72 ± 38.09	0.32
HDL-C (mg/dL)	52.29 ± 12.78	47.68 ± 10.99	< 0.0001^a^
LDL-C (mg/dL)	112.63 ± 30.79	119.34 ± 33.67	0.09
TG (mg/dL)	140.25 ± 62.48	143.3 ±74.5	0.44
BChE absolute activity (KU/L)	5.1 ± 1.53	4.88 ± 1.04	0.01^a^

BChE: butyrylcholinesterase; BMI: body mass index; HDL-C: high density lipoprotein cholesterol; LDL-C: low density lipoprotein cholesterol; TC: total cholesterol; TG: triglyceride.

^a^ Significant values.

HDL-C was the only fraction that showed a significant decrease; the values of the other biochemical variables (TG, TC, and LDL-C) showed no significant changes (Table 1).

Multiple regression analysis was performed to verify the independent effect of BChE molecular forms (G1, G1-A, G2, G3, and G4), before and after dietetic intervention, on the lipid profile (TC, TG, HDL-C, and LDL-C) and anthropometrical parameters (BMI, WSR, and ASR). Before the intervention, G2 acted independently in the determination of TC levels and WSR. After the intervention, G2 showed an independent effect on ASR. G1-A showed an independent effect on WSR before the intervention and on TC levels after. G1 showed an independent effect on WSR before the intervention (Table 2).

**Table 2:** Results from multiple regression analysis

	Dependent variables	Independent variables considered	Confirmed effect	β	P
Before intervention	Lipid profile and anthropometrical parameters	G1, G1-A, G2, G3, and G4	G2 on TC and WSR	0.26 and 0.51, respectively	0.02 and 0.04, respectively
			G1 on WSR	0.56	0.003
			G1-A on WSR	0.58	0.001
After intervention	Lipid profile and anthropometrical parameters	G1, G1-A, G2, G3, and G4	G2 on ASR G1-A on TC	0.35 0.28	0.001 0.01

ASR: abdominal-stature ratio; TC: total cholesterol; WSR: waist-stature ratio.

The phenotype frequencies of CHE2 C5- and CHE2 C5+ were, respectively, 90.84% ± 2.53 and 9.16% ± 2.52. The comparison of mean absolute BChE activity between CHE2 C5+ and CHE2 C5- phenotypes before the intervention did not show a difference in the sample of studied women. After the intervention, only the CHE2 C5- phenotype suffered a significant decrease, and CHE2 C5+, besides not showing a decrease, presented approximately 23% higher activity than CHE2 C5- (Table 3).

**Table 3:** Comparison of BChE activity (± SD) between CHE2 C5- and CHE2 C5+ phenotypes before and after dietary intervention (a) and between the same phenotype in response to dietary intervention (b)

Phenotype	Before	After	(P)^b^
CHE2 C5-	5.03 ± 1.56	4.78 ± 1.0	0.037*
CHE2 C5+	5.74 ± 2.14	5.89 ± 1.24	0.69
(P)^a^	0.13	0.002*	

^*^ Significant values.

Before the intervention, there was no difference between the RA of G4 of CHE2 C5+ and of CHE2 C5- individuals. After the intervention, CHE2 C5+ individuals maintained their RA of G4, while it decreased in CHE2 C5- individuals (Table 4). The analysis of total BChE activity also showed that the CHE2 C5- phenotype was the only one affected by the dietetic intervention (Table 3), reinforcing that there is a different biochemical interaction in the CHE2 C5+ phenotype regarding the effects of an energetic restriction diet intervention. On the other hand, the RA did not show a significant decrease after the intervention or a difference between the CHE2 phenotypes for the G2, G1-Alb, and G1 bands (Table 4), suggesting that the dietetic intervention was not effective on these specific bands.

**Table 4:** Comparison (P) of BChE bands’ relative activity (KU/L) before and after dietetic intervention and between CHE2 C5+ and CHE2 C5- phenotypes

	CHE2 C5-	CHE2 C5+	(P)
G4 (before)	3.36SD1.25	3.91SD1.86	0.24
G4 (after)	3.07SD0.81	3.89SD1.0	0.006^a^
(P)	0.02^a^	0.97	
G2 (before)	0.55SD0.35	0.56SD0.32	0.93
G2 (after)	0.58SD0.32	0.72SD0.42	0.22
(P)	0.37	0.24	
G1-A (before)	0.72SD0.38	0.79SD0.39	0.6
G1-A (after)	0.71SD0.44	0.73SD0.35	0.92
(P)	0.78	0.37	
G1 (before)	0.46SD0.34	0.36SD0.29	0.41
G1 (after)	0.44SD0.32	0.44SD0.27	0.99
(P)	0.52	0.38	

^a^ Significant values.

## DISCUSSION

Anthropometrically, this 8-week diet was efficient, leading to a reduction of WC, AC, and BMI, however, not enough to decrease the BMI to the normal range, but it was sufficient to decrease the mean plasma absolute BChE activity significantly. These results suggest that there is a significant influence of the diet intake on plasma BChE activity, supporting the role of BChE in lipid metabolism and obesity (15,18,28-30).

It has been suggested that the role of BChE in lipid metabolism could be the hydrolysis of choline esters, which are results of the nonesterified fatty acid metabolism and liver lipogenesis (28-30). Considering that in obesity the increased lipogenesis from carbohydrates leads to hyperlipidemia and to increased BChE activity, it could also explain why the energetic restriction diet, due to the decreased availability of carbohydrates in the organism, decreased BChE activity. In our study, after the dietary intervention, the BChE activity of the obese women became similar to the described mean activity of nonobese women from Southern Brazil (4.68 ± 1.51 KU/L) (31).

The fact that the HDL-C levels decreased after the intervention can be explained by a possible lack of physical exercise (PE) during the dietetic intervention, although this is only a nontestable hypothesis since we do not have systematized data that allow inference of the level of physical activity of the women who composed our sample. The lack of PE may cause an excessive formation of ammonia that leads to fatigue and therefore an even greater decrease of PE habits (32) Possibly, other parameters (TG, TC, and LDL-C) would need a longer or a stronger intervention to decrease their values, in contrast to Silva and cols. (33), who demonstrated a significant reduction of biochemical parameters after a 12-week (PE) intervention in obese adolescents. Considering that both interventions decreased BChE activity, it is possible that a combination of PE and diet could be ideal (33).

The relations confirmed by multiple regression analysis between molecular forms of BChE and anthropometrical parameters and TC before (G2 on TC, β = 0.26 and *p* = 0.02) and after (G1-A on TC, β = 0.28 and *p* = 0.01) the intervention are mainly due to its relation with lipid metabolism, BMI, and WC, as related before and in other studies (34-38). As for the BChE molecular form’s individual effect on enzymatic activity, our results are different from the decreased RA of all BChE bands observed by Silva and cols. (33), probably due to the higher effect and duration of PE intervention. In the present study, only the RA of the G4 band showed reduction after the intervention, probably because the G4 band is the most abundant and therefore responsible for most of the plasma BChE activity.

The phenotype frequencies of CHE2 C5- and CHE2 C5+ are in accordance with the frequency of CHE2 C5+ in a population sample from Southern Brazil (10.3%; SD = 0.60; *p* = 0.89) (16). Previously, the phenotype CHE2 C5+ was associated with increased BChE activity but with a lower weight (16) and BMI (18). The resistance of CHE2 C5+ individuals to BChE activity decreased after the intervention compared to CHE2 C5- individuals, which is in accordance with other works, which showed increased BChE activity in the CHE2 C5+ phenotype (15,18) and also suggest that the intervention did not affect the complex G4 associated with the *CHE2* protein, highlighting the importance of understanding the biochemical interactions of the CHE2 C5+ phenotype and BChE activity.

Among the limiting factors of our study was the relatively small sample size, which diminished the power of the study and may have contributed to the nonidentification of effects, especially of smaller magnitudes. Another limiting factor may be that we did not combine dietary intervention with an exercise program to maximize some results. However, our study presents original and specific data about the CHE2 C5 phenotype’s influence on the dietetic intervention’s effect on total and relative BChE activity. Other studies are still required for a full understanding of the interaction between the *CHE2* locus products and BChE concerning the response to metabolic changes. These data helped to increase the knowledge about the role of BChE in obesity, showing how much the levels of enzyme activity are influenced by diet, independent of BMI entering in normal threshold.

In conclusion, after the 8-week diet, a decrease in the BMI, WC, AC, and HDL-C was observed, establishing a relation between the dietetic intervention and the decrease of plasma absolute BChE activity. However, CHE2 C5+ individuals were resistant to this decrease in enzyme activity, maintaining a high level of the RA of G4, which provides the major portion of BChE’s active form, suggesting that the *CHE2* locus holds a strong biochemical relation with increased BChE activity, maintaining its elevated level even after the energetic restriction.

Acknowledgements: *Diagnósticos do Brasil* (DB) clinical laboratory performed the automated measurements of biochemical parameters.

Financing statement: grants and scholarships were received by Araucaria Foundation and *Coordenação de Aperfeiçoamento de Pessoal de Nível Superior* (Capes).
